# Heart Failure With Preserved Ejection Fraction and Low B-type Natriuretic Peptide: A Diagnostic Dilemma

**DOI:** 10.7759/cureus.82602

**Published:** 2025-04-19

**Authors:** Tyler Paul, Amin Ur Rehman Nadeem, Syed M Naqvi, Jason Liu, Khaled A Ali

**Affiliations:** 1 Department of Internal Medicine, Chicago Medical School at Rosalind Franklin University of Medicine and Sciences, North Chicago, USA; 2 Department of Critical Care Medicine, James A. Lovell Federal Healthcare Center, North Chicago, USA; 3 Department of Pulmonary Disease, Chicago Medical School at Rosalind Franklin University of Medicine and Sciences, North Chicago, USA

**Keywords:** b-type natriuretic peptide, congestive heart failure, heart failure with preserved ejection fraction, natriuretic peptides, shortness of breath

## Abstract

Heart failure (HF) is referred to as a clinical syndrome caused by an abnormality in the cardiac function/structure of the heart, leading to pulmonary or systemic vascular congestion. B-type natriuretic peptide (BNP) is particularly useful for ruling out heart failure when levels are low due to its high negative predictive value. On the other hand, BNP has reduced specificity for heart failure with preserved ejection fraction (HFpEF) due to increased clearance and enhanced degradation of natriuretic peptides (NP) which contribute to lower BNP levels. In patients with HFpEF, reduced myocardial wall stress is associated with low BNP, as compared to heart failure with reduced ejection fraction (HFrEF). BNP and N-terminal pro-BNP (NT-proBNP) levels may be in the normal range in chronically treated heart failure patients, especially if younger than 75 years. BNP levels can be lower in HFpEF due to factors like obesity and atrial function. NT-proBNP levels vary by ethnicity, with African American patients exhibiting lower levels, potentially linked to salt-sensitive hypertension and left ventricular hypertrophy. In our case report, the patient presented with classic signs and symptoms of HFpEF exacerbation but had a BNP level that was inconsistent with HF. This signifies the importance of over-reliance on BNP in patients with HFpEF.

## Introduction

Heart failure (HF) is a global health burden and is defined as a syndrome caused by abnormalities in the function and structure of the heart, leading to clinical signs and symptoms [1].

Heart failure with preserved ejection fraction (HFpEF), HF with an ejection fraction of ≥50%, is an underdiagnosed condition, and its prevalence is rising. Despite improved management options, HFpEF impacts approximately 3 million people in the United States. On average, these patients are admitted to the hospital about 1.4 times each year and face an annual mortality rate of roughly 15%. The prevalence of low B-type natriuretic peptide (BNP) in HFpEF varies, often in obese patients, due to reduced natriuretic peptide system activity. The signs and symptoms of HF are related to pulmonary or systemic vascular congestion, which include dyspnea, exercise intolerance, fatigue, peripheral edema, and pulmonary crackles [2].

The diagnosis of HFpEF is a challenge, often requiring a combination of echocardiographic criteria, natriuretic peptide levels, and clinical scoring systems such as the HFA-PEFF (Heart Failure with Preserved Ejection Fraction - Pre-test Assessment, Echocardiography, and Functional Testing) or H2FPEF (Heavy, Hypertensive, Atrial Fibrillation, Pulmonary Hypertension, Elder, Filling Pressure) scores [3].

Approximately 33% of HFpEF patients have normal natriuretic peptide (NP) levels. BNP has reduced specificity for HFpEF due to increased clearance of BNP and NT-proBNP, as well as enhanced degradation, which contributes to lower BNP levels. In the same way, there is reduced myocardial wall stress in patients with HFpEF as compared to heart failure with reduced ejection fraction (HFrEF), leading to falsely low BNP levels [4].

A list of differentials may include pulmonary diseases (e.g., chronic obstructive pulmonary disease, pulmonary hypertension), renal dysfunction, and anemia, all of which may present with similar symptoms [5].

Recent guidelines recommend a combination management approach for HFpEF, including the use of sodium-glucose cotransporter 2 (SGLT2) inhibitors alongside diuretics for congestion relief (with consideration of mineralocorticoid receptor antagonists and angiotensin receptor-neprilysin inhibitors in select patients) [6].

HF increases the risk of arrhythmias, including atrial fibrillation/ventricular arrhythmias, thromboembolism leading to stroke, hepatic congestion/dysfunction causing malabsorption, muscle wasting, and pulmonary congestion with respiratory muscle weakness [7]. Therefore, early diagnosis of HF and its management are crucial at an early stage.

In our case report, the patient presented with classic signs and symptoms of HFpEF exacerbation but had a BNP level that was inconsistent with HF. This signifies the diagnostic challenges posed due to the discrepancy between clinical presentation and the BNP levels.

## Case presentation

This is a 50-year-old African American female who presented to the emergency department with complaints of worsening shortness of breath on exertion, weakness, palpitation, increasing lower extremity edema, abdominal swelling with discomfort, and orthopnea. The patient also noted a decrease in her urine output. She denied any recent chest pain, cough, fever/chills, or headache. She mentioned that she was taking her diuretics as prescribed. As per the chart review, the patient was noncompliant with her medications and was admitted to the hospital for similar symptoms in addition to hyperglycemia, but she didn't meet the criteria of diabetic ketoacidosis or hyperosmolar hyperglycemic state, two weeks ago, before this admission.

During prior admission, her complaints were worsening shortness of breath on exertion, bilateral leg swelling, weakness, and nausea. The patient had a bilateral duplex ultrasound of both extremities at that time, and deep venous thrombosis was ruled out. Her B-type natriuretic peptide (BNP) was normal (11.2 pg/mL) at that time, and acute coronary syndrome was ruled out by serial ECGs and high-sensitivity troponin. The patient was discharged on diuretics and compression stockings for her leg swelling. 

The patient’s past medical history was relevant for hypertension, hyperlipidemia, heart failure with preserved ejection fraction, uncontrolled diabetes mellitus (DM) type II, and major depressive disorder. On initial physical examination, the patient appeared tachypneic with a respiratory rate of 28 breaths per minute. Her vital signs included blood pressure ranging from 92/50 to 146/86 mmHg, heart rate between 80 and 92 beats per minute, and oxygen saturation between 97%-99% on room air. The patient was afebrile. Her weight was 134 pounds with a body mass index (BMI) of 23. The respiratory exam showed decreased breath sounds in the posterior lung fields bilaterally with bibasilar crackles. No jugular venous distention on examination. Other findings include bilateral pedal edema and generalized anasarca.

**Figure 1:**
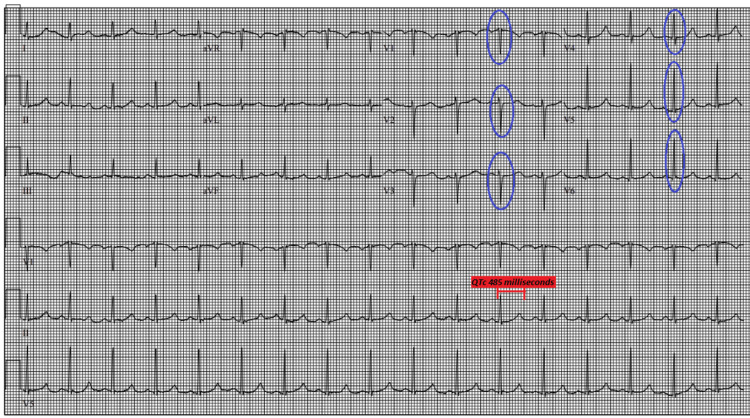
Electrocardiogram showing atypical R-wave progression (shown by blue circles) and long QTc (shown in red), on the day of admission

Initial and follow-up laboratory tests are included in Table 1. An ECG on admission showed poor R-wave progression in anterior leads and prolonged QTc at 485 milliseconds (Figure 1). The QTc prolonging medications were avoided. 

**Table 1:** Labs on admission and on discharge for comparison

Labs	On Admission	On Discharge	Reference Values
Hemoglobin	10.9	11.0	12 - 16 g/dl
White Blood Cells (WBCs)	4.5	4.5	4.3 - 11 ĸ/µL
Platelets	187	217	150 - 400 ĸ/µL
B-type Natriuretic Peptide (BNP)	32	97.8	< 100 pg/mL
Creatinine	0.59	0.65	0.6 - 1.1 mg/dL
Blood Urea Nitrogen (BUN)	17	16	6 - 24 mg/dL
Albumin	3.8	3.7	3.4 - 5.0 g/dL
D-Dimer	1109	-	< 250 ng/mL of fibrinogen equivalent units (FEU)
Aspartate Aminotransferase (AST)	372	31	8 - 33 U/L
Alanine Aminotransferase (ALT)	524	155	4 - 36 U/L
Total Bilirubin	0.6	0.6	0.2 - 1.2 mg/dl
Troponin - High Sensitivity (Troponin-HS)	3	-	<= 34 ng/L
Hemoglobin A1c	>14%	-	Normal < 5.7% Prediabetes >/= 5.7% =/<6.4% Diabetes >6.4%

**Figure 2:**
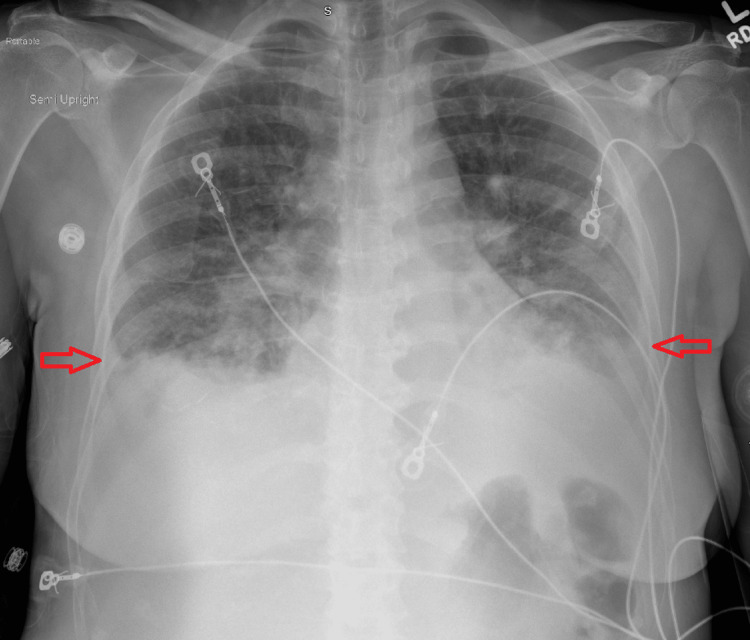
Chest X-ray anteroposterior view showing bilateral pleural effusions on the day of admission (shown by red arrows)

Chest X-ray on admission showed bilateral patchy pulmonary opacities and bilateral pleural effusions (Figure 2). Blood cultures were negative during prior admission. Urinalysis was negative for infection but was positive for glucose > 1000. There were no signs or symptoms of infection during this admission, so the bilateral patchy pulmonary opacities were less likely due to infectious causes and more likely related to her acute heart failure condition.

**Figure 3:**
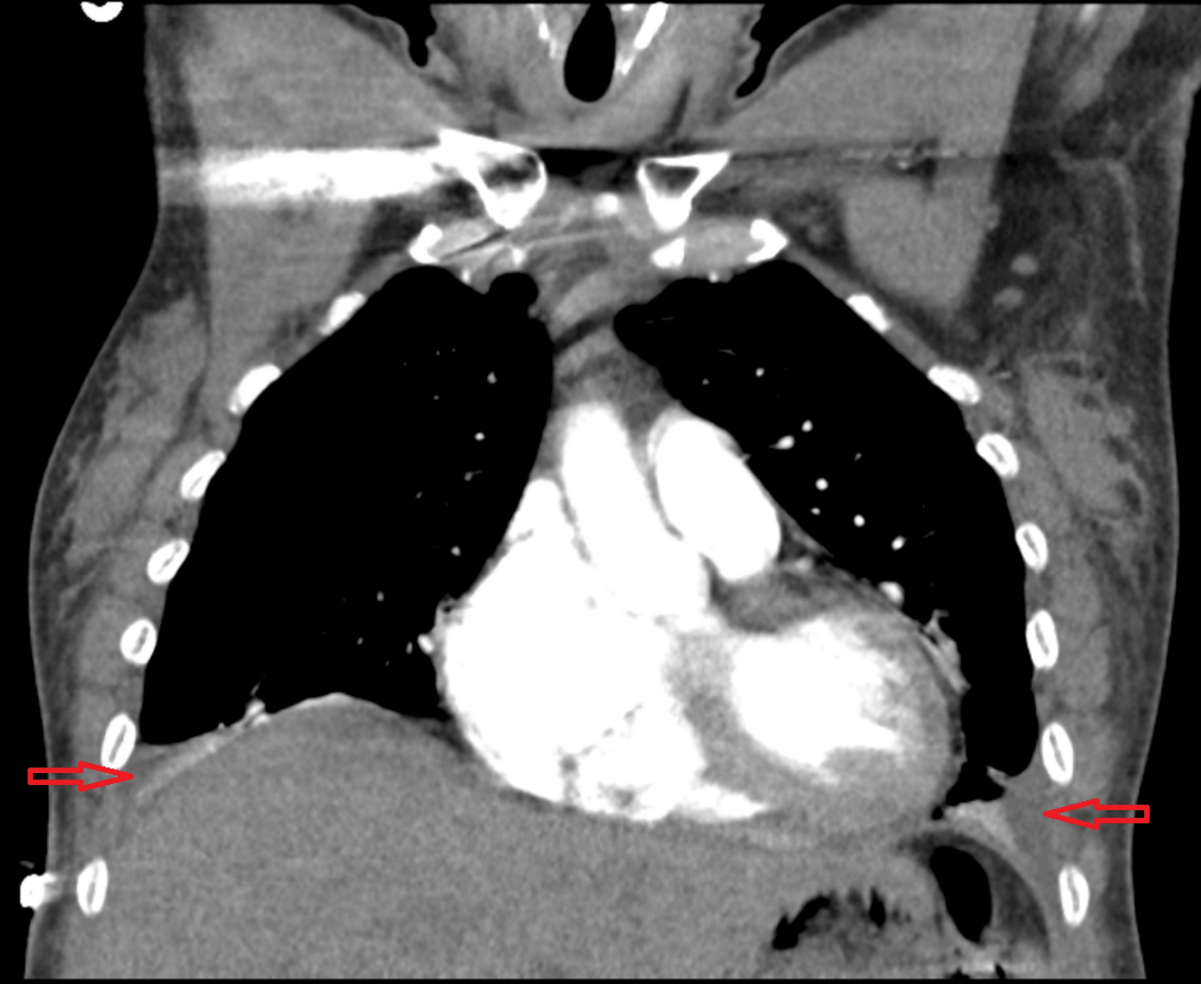
Coronal view of computed tomography (CT) angiogram of the chest showing moderate bilateral pleural effusions on the day of admission (shown by red arrows).

**Figure 4:**
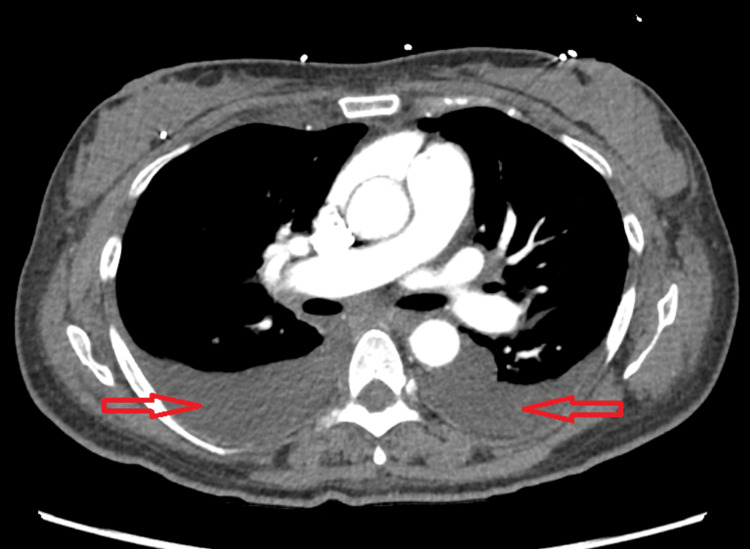
Axial view of computed tomography (CT) angiogram of the chest showing moderate bilateral pleural effusions on the day of admission (shown by red arrows).

Computed tomography (CT) angiography of the chest was performed, due to her palpitations, tachypnea, shortness of breath and elevated D-dimer levels, which ruled out pulmonary embolism but showed moderate bilateral pleural effusions, mild interstitial pulmonary edema, and mild cardiomegaly, which was suggestive of congestive heart failure (CHF) (Figure 3 and Figure 4). Additionally, there were a few scattered lung opacities noted. 

The patient was admitted to the intensive care unit (ICU) due to increased work of breathing, worsening shortness of breath, and increased oxygen requirements. The patient was managed with intravenous bumetanide and oral spironolactone for her acute heart failure exacerbation with preserved ejection function. There was no history to suggest the possibility of negative pressure pulmonary edema or transfusion-related acute lung injury (TRALI). The patient did not have a history of cirrhosis, and serum albumin was in the normal range, essentially ruling out pulmonary edema from low oncotic pressure. The acute coronary syndrome was ruled out by serial ECGs and high-sensitivity troponin. The transthoracic echocardiogram (TTE) showed a left ventricle ejection fraction of 55%-60%, normal left and right ventricular size and systolic function without regional wall motion abnormalities, left atrium size was 2.93 cm which was normal (normal range, 1.9-4.0 cm) and estimated pulmonary artery systolic pressure (PASP) was normal at 34 mmHg but showed mild concentric left ventricular hypertrophy. The nuclear cardiac stress test was done due to concern for abnormal ECG but found negative for any reversible defect to suggest myocardial ischemia.

The cardiologist recommended treating the patient for acute heart failure with preserved ejection fraction exacerbation. The patient showed clinical improvement in dyspnea, peripheral edema, urine output, and liver function tests over the following several days while on active diuresis, and her oxygen requirement was weaned off.

Her anemia is also considered one of the contributing factors to her shortness of breath. Her blood glucose was elevated in the 300s, which was managed by adjusting her insulin regimen. Her elevated liver transaminases were believed more likely to be related to congestive hepatopathy secondary to congestive heart failure (CHF), but we did a comprehensive workup, including the CT abdomen/pelvis, which showed cholelithiasis, gallbladder wall thickening/edema, mild hepatomegaly from periportal edema, new mild diffuse anasarca, a small amount of pelvic free fluid, 1.6 cm indeterminate liver lesion and the right upper quadrant ultrasound showed mild hepatomegaly, 1.6 cm nodule, cholelithiasis with diffuse gallbladder wall thickening, and pericholecystic edema. Her hepatitis panel was normal, and her coagulation profile was within normal limits. Her atorvastatin was held on admission but resumed once her liver transaminases normalized. The patient was being followed with the gastrointestinal clinic as an outpatient after discharge.

It is interesting to note that the patient had a BNP in the normal range but slightly increased as compared to the value on admission in at the time of discharge when her symptoms and radiologic findings improved (Table 1), likely related to the patient's clinical dynamics of heart failure (HF) management such as due to the mobilization of third-space fluids. Moreover, fluctuations in BNP levels can also be influenced by non-cardiac factors such as renal issues, age, and other comorbid conditions [1].

**Figure 5:**
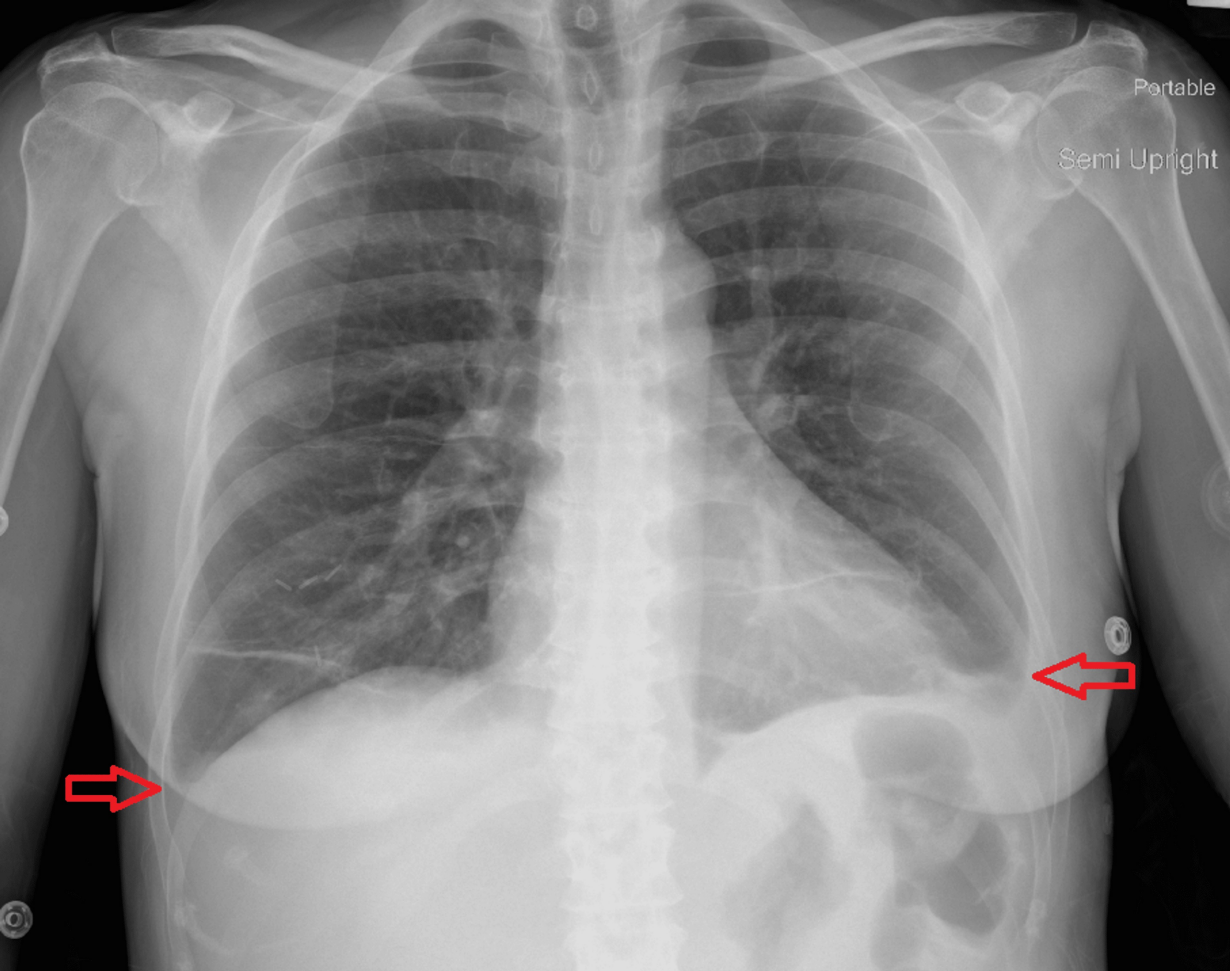
Chest X-ray anteroposterior view showing interval decrease in bilateral pleural effusions on the day of discharge - Day 6 (shown by red arrows)

A follow-up chest X-ray showed the resolution of pulmonary congestion and improvement in bilateral pleural effusions (Figure 5).

The patient was started on oral diuretics, including bumetanide and spironolactone, and empagliflozin regimen, and the patient was continued on her home dose of lisinopril. The patient was emphasized regarding compliance with her medications. She was discharged home with instructions to follow up with her primary care physician, cardiologist, and a diabetic educator. Upon cardiology follow-up, she was also started on a beta-blocker.

## Discussion

Heart failure (HF) is a clinical syndrome resulting from cardiac structural and/or functional abnormality, characterized by clinical symptoms supported by elevated natriuretic peptide (NP) levels with or without evidence of systemic or pulmonary congestion [1]. B-type natriuretic peptide (BNP) has reduced specificity for heart failure with preserved ejection fraction (HFpEF), for example, in obesity, increased clearance of BNP and NT-proBNP via the natriuretic peptide clearance receptor (NPR-C), and enhanced degradation by neprilysin contribute to lower circulating levels. In HFpEF, reduced myocardial wall stress/ventricular dilation compared to heart failure with reduced ejection fraction (HFrEF) results in lower BNP levels [6].

BNP is particularly useful for ruling out heart failure when levels are low due to its high negative predictive value. The use of angiotensin-converting enzyme inhibitors/angiotensin-II receptor blockers and diuretics lowers BNP levels, which signifies its role in monitoring heart failure patients. For example, in chronically managed heart failure patients younger than 75 years, BNP and NT-proBNP levels may be in the normal range, i.e., <100 pg/mL and <125 pg/mL, respectively [8]. 

Natriuretic peptides (NPs) increase natriuresis and cause vasodilation in response to myocardial stretch secondary to myocardial hypertrophy/fibrosis [8]. In this case report, the patient exhibited classic signs and symptoms of congestive heart failure (CHF) but had a BNP level of only 32 pg/mL - a value inconsistent with CHF. This discrepancy highlights the diagnostic challenges posed when clinical presentation and BNP levels do not align. Kondo et al. also reported that 23.2% of symptomatic CHF patients with a left ventricular ejection fraction of ≥45% had low NT-proBNP levels (<125 pg/mL). These individuals were often younger, less frequently male, and had a higher body mass index [9].

Additional studies also emphasize the role of other factors influencing natriuretic peptide levels. Diabetes mellitus impacts BNP levels through altered metabolism and clearance mechanisms. For example, NT-proBNP levels are inversely related to obesity, especially in the context of insulin resistance, commonly seen in diabetic patients [10]. Gupta et al. found that NT-proBNP levels vary by ethnicity, with African American patients exhibiting lower levels, potentially linked to salt-sensitive hypertension and left ventricular hypertrophy [11]. Macheret et al. demonstrated that patients with advanced hypertension may fail to exhibit compensatory increases in NP levels [12].

In this case, the patient was African American and had diabetes mellitus and left ventricular hypertrophy, both of which are associated with lower BNP levels. Despite this, the patient responded well to treatment for decompensated CHF, further supporting evidence that some patients may have relative or absolute deficiencies in NP levels.

When managing CHF in patients with low BNP levels, clinicians should prioritize comprehensive clinical assessments and diagnostic evaluations, as BNP levels alone may be insufficient to guide therapy. A thorough physical examination should assess for signs such as jugular venous distension (indicative of increased central venous pressure), an S3 heart sound (suggestive of volume overload), abdominal distention (indicative of ascites), and bilateral lower extremity swelling (a common sign of fluid retention). Imaging studies such as chest X-rays, which can identify pulmonary congestion, cardiomegaly, and pleural effusions, and computed tomography (CT) scans of the chest for detailed pulmonary and cardiac evaluation are recommended. Point-of-care ultrasound (POCUS) can assess for B-lines (pulmonary edema), pleural effusions, and cardiac function. Transthoracic echocardiography (TTE) is essential for evaluating left ventricular ejection fraction, diastolic function, valvular abnormalities, and pulmonary artery pressure [6]. Selected patients may benefit from cardiac stress testing to evaluate cardiac ischemia and functional capacity [13]. In complex cases, invasive hemodynamic monitoring (e.g., right heart catheterization) can provide critical data on pulmonary capillary wedge pressure (PCWP) and cardiac output [14].

Guidelines from the American College of Cardiology, American Heart Association, and Heart Failure Society of America emphasize the importance of clinical evaluation and imaging in diagnosing heart failure, particularly when BNP levels are inconclusive [6].

## Conclusions

When assessing patients with suspected heart failure, particularly those with heart failure with preserved ejection fraction and significant comorbidities (such as diabetes mellitus and advanced hypertension/left ventricular hypertrophy), clinicians ought to consider the potential for atypical presentations with low B-type natriuretic peptide levels. A comprehensive approach, including a focused physical exam (such as jugular venous distension, an S3 heart sound, abdominal distention, and bilateral lower extremity swelling), relevant imaging (such as chest X-rays, computed tomography (CT) scans of the chest, point-of-care ultrasound) and other diagnostic tools such as transthoracic echocardiography and hemodynamic parameters, is more likely to enhance patient outcomes by ensuring accurate diagnosis and appropriate management.
